# COVID-19 Pandemic-Related Arguments in Polish Civil Litigation

**DOI:** 10.1007/s11196-021-09875-1

**Published:** 2022-01-05

**Authors:** Anna Piszcz

**Affiliations:** Faculty of Law, University of Białystok, Białystok, Poland

**Keywords:** COVID-19 pandemic, Litigation, Arguments, Ordinary courts judgments portal, Written justifications, Court rulings, Legal submissions

## Abstract

The aim of this paper is to analyse the legal record on civil litigation from mid-March 2020 to mid-July 2021 and examine COVID-19 pandemic-related arguments in a sample of litigated cases heard in Polish courts, more precisely 41 cases. In an attempt to establish the number and types of court cases in which such arguments have been raised, the population of individual case records was accessed electronically from the Ordinary Courts Judgments Portal (Pol. *Portal Orzeczeń Sądów Powszechnych*). The analysed research material consists of texts of written justifications published along with rulings of courts of the first instance in the Portal, except for texts regarding criminal cases and widely understood labour cases. This paper refers to certain theoretical aspects of argument and argumentation. Then, it sheds light on the use of COVID-19 pandemic-related arguments by the parties involved in litigation—as reported by the courts in written justifications—considering, amongst others, whether those arguments were found convincing by the courts. Based on a survey of relevant cases, an attempt was made to identify categories of COVID-19 pandemic-related arguments of the parties involved in litigation, raised in their legal submissions. Also a look into the tendencies in this regard was taken to see whether any patterns emerge and it is possible (or not) to discern different trends in the analysed phenomena. The point of the analysis in this article is both descriptive and normative.

## Introduction

It may generally be assumed that COVID-19 has an impact on civil justice within each jurisdiction, even though this impact varies across them and over time, especially as regards the degree of pivoting to remote hearings, restricting the presence of parties and witnesses as well as digitising paperwork ([15], p. 2). A 'new normal' has only just begun to arrive in the Polish courts in relation to remote hearings, and it is not possible to predict whether it will take us to a special country-wide software enabling identification of the participants and ensuring safe connection as well as safe transmission of data ([15], p. 164). There is also significant concern at the effects that technology can have on the trial process—how can the mode of the hearing affect its outcomes? To the best of my knowledge, no such research has occurred in Poland so far, even though in England and Wales the research has been completed regarding the impact of COVID-19 measures (in particular remote hearings) on the civil justice system [5], and its findings are significant, and (perhaps) can be generalized. Although on the one hand the majority of respondents felt that the mode of the hearing had affected neither the way arguments were understood nor the quality of submissions and of the decision-making process itself, on the other hand some respondents were uncertain whether the judge had understood the arguments they had made ([5], p. 47).

However, this article shall not focus on the impact of the mode of the hearing on the way arguments were understood by the judge. The aim of this paper is to examine only widely understood COVID-19 pandemic-related arguments, irrespective of the mode of the hearing, in a sample of litigated cases heard in Polish courts. In an attempt to establish the number and types of court cases in which COVID-19 pandemic-related arguments have been raised, the legal record on civil litigation from mid-March 2020 to mid-July 2021 (16 months) was analysed. The population of individual case records was accessed electronically from the Ordinary Courts Judgments Portal (Pol. *Portal Orzeczeń Sądów Powszechnych*) [22].

This paper refers concisely to certain theoretical aspects of argument and argumentation. Then, the paper sheds light on the use of COVID-19 pandemic-related arguments by the parties involved in litigation—as reported by the courts in written justifications (grounds of judicial decisions)—considering, amongst others, whether those arguments were found convincing by the courts. Based on a survey of relevant cases, an attempt was made to identify categories of COVID-19 pandemic-related arguments of the parties involved in litigation, raised in their legal submissions. Also a look into the tendencies in this regard was taken to see whether any patterns emerge and it is possible (or not) to discern different trends in the analysed phenomena. The point of the analysis in this article is both descriptive and normative.

## Arguments in Litigation

Argumentation as an operation is a communicative action. In other words, argumentation is a part of communication, whereas—in general—communication is accompanied with argumentation. Argumentation theories typically reiterate the view that arguing is aimed at persuading or convincing audiences or interlocutors to whom it is addressed of the value of the theses for which it seeks assent. It is not scrutinised here how argument and arguing are defined by particular argumentation theories such as classical rhetoric, neo-rhetoric, the 'pragma-dialectics' and others [8]. It is assumed that an argument (understood widely also as what people take to be reasons why something is true or something should be done (cf [4], part 2)) has the function of demonstrating the validity or non-validity of the theses. Parties to the proceedings present the theses and the judge evaluates whether their arguments manage to show the validity of the claim(s) they are meant to support. The parties exert through speech a persuasive action on the judge. However, no unanimity exists—in argumentation studies—on the legitimacy and treatment of emotion ([19], p. 170). Nevertheless, assent is considered an unreliable indicator of the goodness of argumentation because it can be given or denied for numerous reasons, including irrational ones ([25], p. 274).

Although most theories of legal argumentation are concerned with the justification of legal decisions ([16], p. 285), in this paper a piece of behaviour is examined and described—more precisely, argumentative communication of the parties to the proceedings—as reported by the courts in written justifications. It is assumed that arguments supporting the party's claim(s) described by the court come from this party's submissions, be they written or oral. Even cursory review of their contents draws attention to some recurrent more convincing (strong) and less convincing (weak) arguments. However, an in-depth analysis of all of the facets of argumentative communication of the parties to the proceedings is beyond the scope of this article. Amongst others, aspects of the reliability or credibility of the source providing an argument are not captured ([10], pp. 337–367).

The tools of argumentation theories can be combined with those provided by persuasion research. For the purposes of this article, it is worth to dedicate some space to the latter in the context of the psychology of judicial decision-making. A group of theories in cognitive and social psychology known as dual process theories have been particularly influential in understanding how people think about information when they make judgments, and have largely documented and illustrated, among others, routes to persuasion. The two big dual process theories are S. Chaiken's heuristic-systematic model of persuasion and R. Petty and J. Cacioppo's elaboration-likelihood model, both developed in the 1980s.

Chaiken distinguished between two thought processes (depending upon, *inter alia*, cognitive and motivational aspects) that can be classified as either systematic processing or heuristic processing ([6], pp. 246–266). First, systematic processing involves attempts to thoroughly understand any available information through careful attention, deep thinking, and intensive reasoning (slow processing). Second, heuristic processing involves focusing on salient and easily comprehended cues that activate well-learned judgmental shortcuts (fast processing). So, reasoning is systematic as opposed to heuristic. An analogous distinction in the context of modes of thought was drawn by D. Kahneman who distinguishes between effortless intuition (system one) and deliberate reasoning (system two) ([12], pp. 697–720).

Petty and Cacioppo in their elaboration-likelihood model suggested that there were 'central' and 'peripheral' routes to persuasion, with:

1. The 'central route' (encompassing Chaiken's systematic view of persuasion) representing the processes involved when elaboration likelihood is high, and
2. The 'peripheral route' typifying the processes operative when elaboration likelihood is low ([20], pp. 173–195).

When elaboration likelihood is high ('central route'), issue-relevant thinking tends to be the most direct determinant of the recipient's reactions to the recommendation, whereas when elaboration likelihood is low ('peripheral route'), the more important determinant of persuasion tends to be cues that, although perhaps peripheral to the personal merits of the appeal, allow the recipient to attain a reasonable position without diligently considering the merits of the arguments for the specific recommendation.

There are two prominent models of reasoning and judgment—top-down and bottom-up processes—that are applicable also to the judicial domain. While both top-down and bottom-up processes involve systematic processing (as defined by Chaiken's heuristic-systematic model of persuasion), the key difference between the two models relates to the extent to which ideological predispositions bias the entire reasoning process ([3], p. 43). Top-down (deductive) processing represents the most biased reasoning process, where the generic predispositions, perceptions, or theories people bring to a judgment context dictate how they process the new information in front of them. These predispositions provide a lens through which facts of the case, evidence, past precedent(s) and legal doctrine, the arguments in the briefs, oral arguments, and other legal considerations are evaluated and assessed. In contrast to top-down processing, bottom-up (inductive) processing represents the most unbiased process, where objective scrutiny of the information, facts, evidence at hand, and so forth, is involved ([3], p. 44). In the persuasion context, bottom-up processing involves yielding to strong and influential arguments ([3], p. 46).

In the context of judging, it is reasonable to assume that judges engage in systematic (and not heuristic) processing of the facts, briefs, oral arguments, and so forth, when making decisions ([3], p. 43). This assumption is accepted for the purposes of this article. However, judicial decision-making can involve subjective choices, so in practice judges rarely engage in pure systematic processing (bottom-up reasoning). It is worth referring to scientific research demonstrating that judges are likely to be vulnerable to many of the ordinary cognitive and social biases pervading human cognition (even though they may be able to impressively suppress bias in some circumstances) ([2], pp. 4, 24), and jury members are likely to use cognitive schemas, such as e.g. stereotypes ([17], pp. 173–180).

## Empirical Study: Methodology and Sample of Analysis

The sources were collected through desk research which meant not only reviewing previous literature but also analysing published court rulings derived from the database chosen after a preliminary search in databases available at the time of research. The analysed research material consists of texts of written justifications published along with rulings of courts of the first instance in the Ordinary Courts Judgments Portal (Pol. *Portal Orzeczeń Sądów Powszechnych*).

In Poland, there exist a few systems and hierarchies of state courts (as well as the State Tribunal and the Constitutional Tribunal^1^) with hugely varied procedures and differentiated structures. The Polish justice system consists of the Supreme Court, administrative courts (including the Supreme Administrative Court), military courts and ordinary courts, i.e. 318 district courts (Pol. *sądy rejonowe*), 46 regional courts (Pol. *sądy okręgowe*) and 11 courts of appeal (Pol. *sądy apelacyjne*).^2^ Their anonymised decisions are posted in an electronic form in official databases, that is—accordingly—the Database of Supreme Court Decisions, the Central Database of Administrative Court Rulings and the Ordinary Courts Judgments Portal (there is no such database for military courts). Only the Ordinary Courts Judgments Portal is relevant for this research.

The Ordinary Courts Judgments Portal is an official platform in which anonymised decisions of ordinary courts from all over the country are posted in an electronic form. It is a regularly updated source of published decisions with written justifications (grounds of judicial decisions). The Portal is a part of the activities of the Polish Minister of Justice (public administration body), which increases its credibility and value. It is an open access and free database. New functionalities are added thereto, such as a good judgment indexing system. It is worth emphasising that a panel of judges is working on the Portal.

Furthermore, individual courts publish their decisions also on their own webpages. However, the Portal collects decisions of all ordinary courts, as opposed to websites publishing judgments of individual courts. As a rule, each court decision published on their webpage, is also published in the Portal. Even though the Portal sometimes omits a judgment from local databases, the review of all the databases of individual courts would be impossible without the use of a special computer programme.

There are also open access and free private databases such as the Journal of Judgments and Court Announcements EBOS.PL (Pol. *Dziennik Wyroków i Ogłoszeń Sądowych EBOS.PL*) and the Judgments Analysis System SAOS (Pol. *System Analizy Orzeczeń Sądowych SAOS*), but they have been founded on a private initiative and are based on official websites with court judgments. Last but not least, there are also 'closed' commercial databases: Legalis (by CH Beck) and Lex (by Wolters Kluwer). They are based on official databases and only from time to time are supplemented with unpublished judgments. They are hardly able to add more value for the conducted research. Therefore, the Ordinary Courts Judgments Portal is considered the best choice for this research.

The usage of mere written justifications is one of the limitations of this research. First, in the case of interrogations of the parties and oral arguments, it does not allow to examine paralinguistic aspects of argumentative communication of the parties and judges. For example tone of voice or emotional states expressed in faces of the participants of the court sitting cannot be adequately transferred in a written context, and it is impossible to recognise whether the parties were engaged in reasoned argument, reporting, explaining, or rather quarrelling, disputing. Second, in the case of the parties' written submissions, written justifications are only secondary (indirect) sources of the information. It can be seen from written justifications what the parties stated, but the parties' linguistic style, i.e. how they wrote and what the stylistic component of the argument was [with regard to stylisation of written justifications of judicial decisions, cf. [14], part 2] is not known from these sources. In this research the information based on argument quality is processed—as reported by the courts in written justifications. However, the acceptance of these limitations allows for preventing very protracted research activities in COVID-19 pandemic times.

Rulings adopted in the following types of cases are not addressed in this research: widely understood criminal cases (with letter K or W in case reference numbers), labour cases (with P in case reference numbers), and cases concerning social security law (with U in case reference numbers). The paper is focused on narrowly understood civil litigation (including family cases and in particular alimony cases). Only these types of cases are addressed in this study, since in Poland they are the most adversarial in nature, whereas the court activities *ex officio* are to be an exception. Each party to the proceedings presents arguments and evidence in support of their version of events to the court.

Moreover, court orders (Pol. *postanowienia*) are not addressed. Also rulings of courts of the second instance (with letters ACa or AGa in case reference numbers) are not addressed in this research.

On the 16th of July 2021, the search engine of the Portal shows 336 items, when the key word 'pandemic' (Pol. *pandemia*) is entered. For the sake of clarity it must be noted that the Portal does not cover any and all judicial decisions simply because their publication in the Portal is not mandatory. As a result, whereas some Polish courts publish (almost) all decisions, the others hardly see the need to publish them at all [11]. Therefore, it must be acknowledged that there are also limitations of this research resulting from the limited number of published judicial decisions. After the exclusion of the above-mentioned types of rulings plus the ones regarding past pandemics, such as influenza and African swine fever (ASF), there are 111 rulings of courts of the first instance left for analyses. On the same date as above, the search engine of the Portal shows 10 rulings of courts of the second instance, when the key word 'pandemic' (Pol. *pandemia*) and letters 'ACa' (seven items) or 'AGa' (three items) are entered. Only five of them were handed down as a result of appealing the rulings of courts of the first instance given after the outbreak of the pandemic. However, none of the appealed rulings appeared on the previously mentioned list of 336 items containing the key word 'pandemic'. Therefore, those rulings of courts of the second instance were not scrutinised. They do not contain any assessment of the COVID-19 related arguments raised in cases analysed by this study and, as such, are irrelevant for this research. This search result leads to additional conclusion. The period of up to 16 months (in the cases of rulings of courts of the first instance issued at the beginning of the period from mid-March 2020 to mid-July 2021) is too short to find a ruling of the court of the second instance published in the Portal. The duration of civil proceedings in Poland is considerable. After the oral presentation of a ruling of the court of the first instance, parties have seven days to apply for written justification. Courts have two weeks to write it (also the court of the second instance), but this period can be prolonged. After the delivery of written justification, the party has two weeks to lodge an appeal. If we add time of deliveries, the very appealing of the ruling can take around two months.

Going back to 111 rulings of courts of the first instance found in the Portal, it should be emphasised that the majority of them (58) were adopted in alimony cases (letters RC in case reference numbers). Eight of them were issued in commercial cases, mainly with claims for payment, but also one case with claim for declaratory relief and one case with claim for dissolution of the company (letters GC in case reference numbers). And 45 of them were adopted in the other civil cases (letter C in case reference numbers). They included: claims for payment (also cases with banks as plaintiffs), claims for damages and/or other compensatory redress, claim for injunctive relief, claim for the release from the execution, claim to cancel the enforceability of the enforcement title, claim to repeal a resolution of the housing community (housing cooperative), claim to change the underlying legal relationship (claim for a reduction in rent).

70 of these 111 rulings contain at least one sentence including the word 'pandemic'; however, no concrete COVID-19 pandemic-related arguments were derived therefrom. As a result, in sum, 41 out of 111 rulings were analysed in details.

## Analysis of the Results

This part of the article first discusses the types of arguments. In the research, an attempt was made to 'code' the reported arguments for being part of the distinguished types of arguments and—based on the number of the arguments—to provide detailed statistics for individual types of the reported arguments. Second, in this part of the paper, the characteristics of the reported arguments is provided. As such, this part of the article aims to highlight the contents of the reported arguments. Last but not least, it provides an overview of the trends in COVID-19 pandemic-related argumentation.

### Types of the Arguments

Leaving aside semantic and logical connections, this article refers to metaphors of sword and shield. Depending on a specific case, COVID-19 pandemic either is sufficient to provide a plaintiff with a sword, or may provide a defendant with a more or less effective shield. From this point of view, arguments can be schematically classified as follows: (1) arguments in support of the plaintiff's case (sword arguments) (2) arguments in support of the defendant's case (shield arguments). This can be seen as being quite simplistic, as it does not account for the classification that covers a 'yes duty' as a sword argument as opposed to a 'no duty' as a shield argument and, depending on the context, certain variations (cf. [25], p. 741). In the case of this classification, the argument of a parent suing a child for waiving or reducing child support payments would be a shield argument, and not a sword argument, whereas the child's counterargument would be a sword argument, and not a shield argument. However, the simplistic classification may be useful for the purposes of this paper. Overall, there were 20 sword (plaintiffs') arguments, including 13 strong ones, and 21 shield arguments (defence arguments), including 15 strong ones.

It should be highlighted in this respect that, from time to time, the written justifications are quite vague, and in such instances it is considered they leave it to a researcher to determine—to the best of her/his knowledge—where to draw the line when specific arguments do not fit neatly into the above mentioned categories of arguments (e.g. it is unclear if a specific communication came from the party or the court *ex officio*).

Arguments can also fall into two categories: (1) 'primary' arguments and (2) counterarguments. In this case, it is assumed that argument involves two parties (pro and con) exchanging reasons on an issue ([1], p. 2). In legal proceedings, the parties present their standpoints and arguments, but also they formulate their doubts and critique with regard to the claims and arguments of the other party ([9], p. 223). Quite often, COVID-19 pandemic provided the defendant with the effective counterargument to the 'primary' argument of the plaintiff (the latter not necessarily related to COVID-19 pandemic). Typically, if in the alimony case, the plaintiff claimed the increase of support payments because of the increase of her/his expenses due to the pandemic, the defendant's counterargument might be the loss of employment or adverse changes to income or expenses because of the pandemic (see part 4.2.1 below).

Last but not least, arguments can be broadly divided into: (1) substantive arguments related to the party's claim, and (2) procedural arguments related to the course of the proceedings (including taking evidence or parties' presence at the court sitting) or the legal costs. COVID-19 pandemic-related procedural arguments only appeared in five cases.

### Characteristics of the Arguments

In this part of the paper attention turns to the characteristics of the reported arguments. First, the contents of the reported arguments are presented with the emphasis being on their key elements allowing to classify them ('overview') rather than their details ('scrutiny'). These arguments, as will be revealed below, can be described as the financial arguments with regard to: adverse changes to the party's income, changes to the party's expenses, loss of employment or opportunities for getting into work, as well as the arguments related to mental health and/or physical health, certain restrictions to citizens' rights due to the pandemic and related administrative solutions. The references to general life experience with regard to the pandemic are also mentioned. Second, this part of the paper provides an overview of the trends in COVID-19 pandemic-related argumentation.

#### Content of the Arguments

Due to the COVID-19 pandemic, many individuals have experienced financial hardship (financial crisis). The majority of the analysed cases (brought because of the pandemic or other circumstances) involve pecuniary claims of a varied nature. Therefore, the analysed justifications contain mainly arguments of a financial character. First, these arguments focus on adverse changes to the party's income (group A):

1. Worsening economic situation during the pandemic:
  - Accepted—as the plaintiff's argument (ref. no. III RC 138/20, District Court in Świnoujście, where due to the pandemic, the plaintiff is not currently renting her flat to anyone and her partner receives a lower salary; ref. no. III RC 885/18, District Court in Toruń; ref. no. VI RC 215/20, District Court for the Capital City of Warsaw in Warsaw; ref. no. I C 259/19, Regional Court in Łódź) or as the defendant's argument (ref. no. I C 483/19, District Court in Wąbrzeźno; ref. no. V GC 1261/20, District Court in Toruń);
  - Rejected; a student (the plaintiff) would be able to give private lessons, e.g. in English, in history etc.; all the more so because during the pandemic, private lessons are given online (ref. no. VI RC 144/19, District Court for the Capital City of Warsaw in Warsaw);
2. A pay cut:
  - Accepted—the defendant's argument (ref. no. I C 121/20, District Court in Człuchów; ref. no. III RC 122/19, District Court in Zduńska Wola; ref. no. III RC 412/19, District Court in Otwock);
  - Rejected; the plaintiff claimed a compensation from an insurance company; in the Court's opinion, the state of the pandemic is a transitional period and reducing wages in such particular times to 80% is common for most employees and is not related to their health condition (ref. no. I C 136/18, Regional Court in Sieradz);
3. No or almost no income:
  - Accepted; in the first case, a hairdresser (the defendant) did not have any income in April 2020 when hairdressing salons were closed in Poland (lockdown); due to the pandemic and the lack of clients, she works two hours a day (accepted in: ref. no. III RC 100/19, District Court in Głubczyce)—in the Court's opinion, her premises (with the equipment) where she operates a hairdressing salon constitute a certain property value, but she should not be expected to sell this property, because the conducted business activity is in line with her education, and until the pandemic hit, it gave her a small but stable income; in the second case, a first-year student of administration (the plaintiff) has no profession; during the lockdown, she cannot continue irregular jobs in places such as restaurants, shops with clothes/shoes in shopping galleries; however, due to the specificity of extramural studies (held at weekends and, in the pandemic times, remotely as a rule), the Court advised the defendant to adapt her housing plans to her real financial possibilities and consider returning to the mother's house (ref. no. III RC 94/20, District Court in Łowicz);
  - Rejected; a musician (counter-defendant and plaintiff at the same time) claims that, due to the coronavirus pandemic and restrictions related thereto, he does not have any income from artistic activity and he deregistered his business activity; the court only believes that the income decreased, especially that the party's colleague from the band continues to conduct the same activity (ref. no. III RC 172/19, District Court in Pisz);
4. Due to the pandemic, the party could not continue the economic activity (which could bring an income) at all or to the same extent:
  - Accepted in the case of the gastronomic activity (ref. no. III RC 99/20, District Court in Giżycko; ref. no. I C 957/20, District Court Poznań-Stare Miasto in Poznań);
  - Rejected, as the defendant invested funds to open a restaurant at a time when it was already known that the state authorities would introduce economic restrictions in connection with the pandemic (ref. no. XIV C 325/20, Regional Court in Poznań) or at the time of the ruling restrictions were not yet in force (ref. no. III RC 355/19, District Court in Grudziądz).

Second, the financial arguments focus on changes to the party's expenses (group B):

1. New expenditures:
  - Accepted; new plaintiff's expenses related to protective masks and disinfectants (ref. no. III RC 122/20, District Court in Świnoujście); an increase in costs of the treatment of the plaintiff's non-healing pressure ulcers, caused by the pandemic besides the inflation (ref. no. XIV C 1532/16, Regional Court in Poznań); unforeseen juvenile plaintiff's expenses, such as food price inflation, utilities price inflation, the purchase and insurance of notebook computers due to the need to study remotely (ref. no. III RC 129/20, District Court in Olkusz); the defendant's (father's) counterargument that the plaintiffs' mother should have tried to borrow computers from the school was rejected by the Court; in the Court's opinion, such possibilities are limited, and, moreover, the defendant, as a teacher, knows best how to apply for such help; he should have been much more involved in matters related to the education of the daughters and could have submitted a relevant application to the school;
  - Rejected; the pandemic outbreak forced a first-year student (the plaintiff whose father died in an accident) to return to his family home and study remotely; he claimed a compensation from an insurance company related to his father's death; in the Court's opinion, his arguments that the one-off high compensation would be used by him to start a business or purchase his own flat are unconvincing; it cannot be believed that he would have experience, background and precise professional plans to the extent that would let him start it; the pandemic does not provide favourable conditions for such activity (the plaintiff's arguments rejected in: ref. no. XII C 1809/19, Regional Court in Poznań);
2. a decrease in maintenance costs—the defendant's argument accepted, as the minor plaintiff stopped attending a kindergarten and stays at home (ref. no. II RC 237/20, District Court in Wągrowiec).

Third, the financial arguments focus on loss of employment or opportunities for getting into work (group C):

1. No opportunities or reduced opportunities for the parties to work and/or expand professional qualifications due to the pandemic:
  - Accepted; parents (here, juveniles' mothers) have partially taken over the tasks related to education (ref. no. III RC 264/19, District Court in Koło; ref. no. III RC 93/20, District Court in Szczytno);
  - Rejected; the defendant was looking for a job in security, but the course was cancelled twice due to the pandemic (ref. no. VI RC 6/20, District Court for the Capital City of Warsaw in Warsaw)—in the Court's opinion, due to the low level of unemployment in Poland, the defendant could successfully start running his own business (even despite the current economic situation related to the pandemic);
2. No opportunities or reduced opportunities for young persons to find a job:
  - Accepted; the defendant as a young person is beginning his career in the professional world; he has recently graduated from university and still, in part, is dependent on his mother (ref. no. I C 1071/18, District Court in Chełmno);
  - Rejected; in the Court's opinion, the defendant as a young person attending an extramural secondary school is able to quickly find suitable employment in the area of the capital city, and in the event of difficulties in contacts with other people, she can also successfully work remotely, which is common during the pandemic (ref. no. VI RC 350/19, District Court for the Capital City of Warsaw in Warsaw);
3. Loss of job, additional job or seasonal job—accepted; in the first case, the defendant lost employment (ref. no. III RC 102/20, District Court in Łowicz); in the second case, the defendant lost all opportunities for additional employment (ref. no. I C 60/20, District Court in Nowy Sącz); in the third case, there are reduced opportunities for the defendant to work overtime, as there are fewer additional hours of work (ref. no. III RC 65/20, District Court in Brodnica); in the fourth case, due to the pandemic in the area of the seasonal workplace, the defendant ceased to undertake seasonal work in Germany and she does not intend to go to Germany, fearing for her own health—in the Court's opinion, her earning opportunities are greatly reduced, if not eliminated (ref. no. IV RC 660/18, District Court in Rybnik).

Another group of arguments were related to mental health and/or physical health, even though some of these arguments incur financial implications (group D):

1. The deterioration of the plaintiff's mental condition:
  - Accepted; the plaintiff claimed a compensation from an insurance company related to his wife's accident; after the accident she is a disabled person (she even does not speak) and stays in a special institution; due to the pandemic, the plaintiff could not visit her; he feels lonely; he lost support from his wife needed so much in a difficult time of the pandemic, where he, due to his age, is at a higher risk of the infection (ref. no. I C 250/19, Regional Court in Łomża);
  - Rejected; the plaintiff filed an application to take an expert opinion evidence (psychiatrist's opinion) proving the deterioration of the plaintiff's mental condition related, *inter alia*, to the restricted access to health care during the pandemic; the application was dismissed, as the Court decided, amongst others, that it did not need proof (ref. no. I C 255/20, Regional Court in Sieradz);
2. Restricted access to treatment—accepted; during the pandemic, getting the National Health Fund treatment is largely difficult, limited or suspended, and waiting times are long (ref. no. III RC 159/19, District Court in Łowicz);
3. The deterioration of the plaintiff's physical condition—accepted; during the pandemic, it is very important for the plaintiff as a young person to play sports, especially that he studies remotely (ref. no. III RC 34/20, District Court in Łowicz).

The last group of arguments regard certain restrictions to citizens' rights due to the pandemic and related administrative solutions (group E):

1. The lack of physical presence:
  - Accepted; the defendant (the housing community) ordered a correspondence vote of resolutions instead of the annual meeting with physical presence of members, due to the pandemic (ref. no. I C 1275/20, Regional Court in Słupsk);
  - Rejected; the party living abroad could not attend the court sitting due to the pandemic and travel restrictions (rejected in: ref. no. III C 250/18, District Court Szczecin-Centre in Szczecin, as the defendant did not prove that she had lived abroad, and in: ref. no. III RC 125/19, District Court in Człuchów, but the ruling lacks justification in this regard);
2. Restricted access to competent offices—rejected; the plaintiff tried to re-register the purchased vehicle, but due to the pandemic, the competent office did not carry out these activities, and he intended to re-register the vehicle later (ref. no. I C 1233/20, District Court in Kłodzko); the Court stated that, in fact, the competent offices had remained closed for some time, but the deadline for re-registration had been extended to 90 days, and the plaintiff had not presented even indirect proof that he had been trying to do so;
3. The issue of access to a sport fields pre- and post-pandemic—rejected; the plaintiffs claimed for ordering the defendant (the commune), to refrain from operating a sports field, which has disturbed the use of the plaintiffs' (neighbouring) real estate due to the noise; the situation has improved due to remote learning and the lack of physical education classes during the pandemic, but the noise may reoccur in the future (the claim unfounded, the argument rejected in: ref. no. I C 767/16, District Court in Ciechanów).

Interestingly, it has been found that, in only two rulings out of the 41, there are references to general life experience with regard to the pandemic. Whereas judges are inevitably influenced by their life's experience, significantly shaped by one's linguistic, socio-economic, cultural, and ethnic background ([23], p. 26), Polish judges seem very careful with general life experience when it comes to the new COVID-19 pandemic. They quite properly drew on general life experience concluding that:

1. During the pandemic, private lessons were given online (ref. no. VI RC 144/19, District Court for the Capital City of Warsaw in Warsaw);
2. The cost of assistance for disabled persons has increased no later than at the beginning of the pandemic in Poland, as the pandemic was a basic factor that, besides the inflation, must have influenced such costs (ref. no. XIV C 1532/16, Regional Court in Poznań).

#### Looking into the Trends

The analysed COVID-19 pandemic-related arguments are based on that during the pandemic, individuals, businesses, organisations etc. have not been able to operate as normal. A tacit assumption underlying this research was that litigants might be facing financial difficulties due to the pandemic. Unsurprisingly, content analysis of the written justifications of court rulings revealed that financial motivations were the most frequent amongst the reported arguments. There were also non-financial incentives and motivations of the parties, however, some of them incurred financial implications.

In analysing research material from the reasoning perspective, it is necessary to determine the quality of an argument, i.e., whether it is viewed as strong (more convincing) or weak (see part 2 above with regard to the tools of argumentation theories). As previously stressed, one of the goals of this research was to check whether the examined COVID-19 pandemic-related arguments were found convincing (strong arguments) by the courts or not (weak arguments). In other words, this article aimed at checking which arguments were considered strong/weak by the judging panels, i.e. which ones were accepted/rejected by them. Overall, there were 28 strong arguments and 13 weak arguments. However, rulings of courts of the second instance (with capital letter A in case reference numbers) are not addressed in this research at all (see part 3 above). As a result, we do not know whether any of the parties appealed the ruling, and if so, on what basis (bases) and whether the arguments that had convinced or, to the contrary, had not convinced the court of the first instance were assessed in the same manner by the court of the second instance (if it already adjudicated the case).

When examining all the arguments from a semantic perspective (where argument picks out a certain class of purely semantic entities that are the outcome of the process of reasoning ([13], pp. 239–259)) and looking for relations of conveyance within them, we can see that the majority of the analysed arguments are organised according to the following scheme: fact *x*'s causing fact *y*, i.e. the fact *y* happened to the party, because the COVID-19 pandemic broke out and if the COVID-19 pandemic were to break out, the fact *y* would happen to the party. They can be correctly classified as a certain type of causal arguments, more precisely causal arguments that proceed from the pandemic as cause to effect (the so-called arguments from cause to effect).

In the analysed sample, the parties' arguments *a contrario*, analogical arguments (arguments from analogy), arguments *a fortiori*, i.e. *a minori ad maius* and *a maiori ad minus* ([7], pp. 202–237]), cannot be found. Interestingly, these types of arguments appear in the courts' reasoning. For instance, the defendant's argument that the pandemic had worsened her economic situation was accepted (ref. no. V GC 1261/20, District Court in Toruń), because 'in the current reality of the global pandemic (…) the lack of contractors was regularly confirmed by other entrepreneurs' (in literature it is also qualified as an argument by classification, which assumes that a generalized conclusion about known members of a class applies to a hitherto unexamined item or example, whereas an argument from analogy often relates an unknown phenomenon to a known comparable case ([25], pp. 616ff)). This type of the Court's argument appeared also where the plaintiff's argument that the pandemic had worsened his economic situation was rejected (ref. no. VI RC 144/19, District Court for the Capital City of Warsaw in Warsaw), because according to '(…) life experience and other cases heard in this Division, during the coronavirus pandemic, private lessons are given online (…)'.

Has the analysis of the contents of the arguments demonstrated inconsistent decision-making with regard to COVID-19 pandemic-related arguments of the parties, both within one court (see arguments A.1.a and A.1.b assessed by District Court for the Capital City of Warsaw in Warsaw), and compared to other courts (in particular arguments in A.1, A.2, A.3, A.4, C.2)? Are its findings entirely surprising? The answer should be in negative. The courts take into account individual situation (as affected by the pandemic circumstances) and variables resulting therefrom that have an impact on the strength of the argument. Therefore, judges may reach different results and judges' choices may differ, even if the circumstances of the cases seem similar at a first glance. It is acceptable provided that justifications offer a clear trace of the hierarchy chosen amongst the argumentative reasons instead of giving no explicit clue to rebuild such a hierarchy (the latter is the case for e.g. ref. no. III RC 125/19, District Court in Człuchów). Such clear justifications make judges' preferences transparent, and, these preferences may become, at least, object of discussion ([18], p. 401).

## Conclusion

The law does not, and cannot operate in a vacuum, instead it operates under socio-economic pressures, including COVID-19 pandemic-related social pressures. The pandemic not only had an impact on the Polish 'law in books' (law-making, contents of the statutory laws), which becomes so complicated that requires more and more legal clarifications when applied by competent administrative authorities ([21], p. 601ff). It also results in that—when applied by courts—judges frequently have to apply existing law to the evolving pandemic circumstances as to which it has not been authoritatively laid down that such law is applicable.

It should come as no surprise that COVID-19 pandemic-related arguments are employed in legal submissions of litigants. The parties involved in litigation frequently make arguments which imply that they have been severely hit by a financial crisis (financial hardship) brought on by the COVID-19 pandemic, putting strong emphasis on worrying financial trends, and thus on shifts in their financial capabilities.

In the first group (A) of the reported arguments (nearly 44% of the analysed sample), the adverse effects of the COVID-19 pandemic in relation to the party's income have been claimed. In this group of the arguments, it was claimed that e.g. due to the pandemic, the party could not continue the economic activity (which could bring an income) at all or to the same extent (A.4). This was the case of the arguments related to the gastronomic activity: (a) accepted in two judgments and (b) rejected in two judgments, where the activity was undertaken—respectively—(a) before the lockdown (and at the time of the ruling restrictions were in force) and (b) at a time when it was already known that the state authorities would introduce economic restrictions in connection with the pandemic or at the time of the ruling restrictions were not yet in force at all. In the other cases of the group A, the arguments ranged from a pay cut (A.2) to other signs of worsening economic situation during the pandemic (A.3, A.1).

In the second group (B) of the reported arguments, the focus was on the changes to the party's expenses that increased (higher or new expenditures—see B.1) or decreased (maintenance costs of a child—see B.2) when comparing pre- and post-outbreak expenses. In these arguments, it is claimed that after the outbreak of the COVID-19 pandemic, numerous costs did not remain unchanged compared to their pre-pandemic levels. This is what occurred for example in relation to the increased spending on food, utilities, treatment, protective masks and disinfectants, the purchase and insurance of notebook computers, but also the decreased maintenance costs in the case where the minor plaintiff stopped attending a kindergarten and stayed at home.

In the third group of the arguments under scrutiny (C), the focus was on the loss of employment or opportunities for getting into work. In these cases, the loss of job, additional job or seasonal job was claimed (C.3). Some arguments that belong to the group C are based on the lack of opportunities or reduced opportunities for the parties to work and/or expand professional qualifications due to the pandemic (C.1). The above-mentioned argument has also been worked out in the context of young persons looking for a job (C.2). This is for instance the case of the defendant who has recently graduated from university (argument accepted—see C.2.a, but see C.2.b).

It should be noted, however, that the wider impacts of the COVID-19 pandemic can also be seen from the analysis of the reported arguments—see groups D and E of the arguments. Within these groups of arguments, non-financial incentives and motivations of the parties appeared, even though quite obviously some of them incurred financial changes.

From the perspective of persuasion research (dual process theories—see part 2 with regard to persuasion) and in the context of judging, it seems that, as a rule, judges engaged in systematic (bottom-up) processing of the reported arguments (and not heuristic processing). It should be noted, however, that biases and confounding factors affecting the results cannot be excluded. For instance, in a group of the arguments related to young persons as litigants, there were both those accepted and those rejected by courts. Within each sub-group, the courts were different, i.e. the courts seated in small cities tended to accept the arguments of young persons (Łowicz, Chełmno—see C.2.a and A.3.a), whereas the District Court for the Capital City of Warsaw tended to reject them (C.2.b and A.1.b). The latter argued—referring also to general life experience as a source—that young persons are able to find a job in the area of the capital city quite easily (e.g. students can give private lessons) and successfully work remotely. It may be that we observe the courts' reasoning which, in fact, is amplified by biases (there are stereotypes among them) and this leads to weakening the argument.

It is not a prevailing rule, however, that all COVID-19 pandemic-related arguments are accepted by courts. During the analysis of the research material with the focus on the reasoning perspective (see part 2 with regard to the tools of argumentation theories), the examined COVID-19 pandemic-related arguments were found moderately convincing. There were 28 strong arguments (that convinced the judging panels and were accepted by them) and 13 weak arguments (that did not convince the judging panels and were rejected by them). It does not necessarily mean, however, that we experience inconsistent decision-making with regard to COVID-19 pandemic-related arguments of the parties, either within one court (A.1.a and A.1.b) or compared to other courts (A.1, A.2, A.3, A.4, C.2). Judges go on to address all the facts of the case, evidence, written and oral arguments, as well as other legal considerations. Only seemingly the impression is given that an inconsistent decision-making exists with regard to identical COVID-19 pandemic-related arguments of the parties. The analysed written justifications of court rulings teach that the devil is very often in the details, and, realistically speaking, one cannot escape these details by adopting an overly simplistic view of the pandemic affected party's situation. Such a tendency, if it were indeed in play, would be manifestly inappropriate given the observance of the principle of truth subject to the adversary nature of civil proceedings in Poland ([24], pp. 32–38).

## Footnotes

1 Its decisions can be found in the Online Portal of the Rulings of the Constitutional Tribunal.

2 Civil cases, amongst others, are dealt with by ordinary courts (and, to certain extent, by the Supreme Court).
